# The Benefits of Vaccination against SARS-CoV-2 during Pregnancy in Favor of the Mother/Newborn Dyad

**DOI:** 10.3390/vaccines10060848

**Published:** 2022-05-26

**Authors:** Daniela-Eugenia Popescu, Cosmin Cîtu, Ana Maria Cristina Jura, Nicoleta Lungu, Dan Navolan, Marius Craina, Alin Semenescu, Florin Gorun, Mihai-Andrei Jura, Valerica Belengeanu, Marioara Boia

**Affiliations:** 1 Department of Obstetrics-Gynecology and Neonatology, “Victor Babeș” University of Medicine and Pharmacy, 300041 Timisoara, Romania; popescu.daniela@umft.ro (D.-E.P.); lungu.nicoleta@umft.ro (N.L.); navolan.dan@umft.ro (D.N.); craina.marius@umft.ro (M.C.); gorun.florin@umft.ro (F.G.); boia.marioara@umft.ro (M.B.); 2 Department of Neonatology, Premiere Hospital, Regina Maria Health Network, 300645 Timisoara, Romania; 3 Department of Neonatology, “Louis Țurcanu” Children Emergency Clinical Hospital Timișoara, 300011 Timisoara, Romania; maciu.cristina@gmail.com; 4 Institute for Advanced Environmental Research (ICAM), West University of Timisoara, 300223 Timisoara, Romania; alin.semenescu@e-uvt.ro; 5 Department of Epidemiology, “Pius Brinzeu” Emergency County Hospital Timisoara, 300736 Timisoara, Romania; jura.mihai@gmail.com; 6 Department of Genetics, Institute of Life Science, Faculty of Medicine, “Vasile Goldiş” Western University of Arad, 310025 Arad, Romania; belengeanu.valerica@student.uvvg.ro

**Keywords:** vaccination, SARS-CoV-2, COVID-19, pregnancy, newborn, spike protein, antibodies, breastfeeding

## Abstract

When the first vaccines against SARS-CoV-2 emerged, pregnant women were excluded from clinical trials, so vaccine recommendations were initially adjourned, with late initiation for this populational category. The present study aims to quantify the serum and breastmilk values of SARS-CoV-2 spike protein antibodies in both the mother and her newborn after complete vaccination during pregnancy. Ninety-one vaccinated patients were included, some of whom presented COVID-19 infection during pregnancy. In the delivery room, venous blood was collected from the mother and umbilical cord blood from her offspring. All samples were processed using the ECLIA (electrochemiluminescence) method. Breastmilk was collected and tested during the third postnatal day. The highest maternal serum values were 19,523 U/mL (detection limit > 0.8 U/mL) and in breastmilk, 206.7 U/mL. Every single newborn had antibody values higher than 0, with a mean serum value (M = 5288.37, SD = 5661.49) significantly higher than 0, *t*(90) = 8.91, *p* < 0.001. Consequently, this study intents to emphasize the importance of vaccination against SARS-CoV-2 during pregnancy. This double kind of neonatal protection, attained by placental and breastmilk transfer, can be accomplished by encouraging vaccination, breastfeeding, bonding, and providing maternal empowerment to participate in her infant’s care.

## 1. Introduction

The COVID-19 pandemic affected the entire world population, with one of the most vulnerable categories being pregnant women, presenting a high risk of contracting and developing the disease, along with their fetus and newborn, respectively.

To this date, the studies conducted have shown that expecting mothers have a higher risk of developing severe COVID-19 symptoms compared with non-pregnant women and an increasingly higher risk of those with pregnancy-induced pathologies such as arterial hypertension, diabetes, obesity, or asthma [1]. The risk of preterm labor and delivery for this category is three times higher, and under these circumstances, vaccination during pregnancy becomes mandatory [2,3].

Many scientists worked together to find a vaccine and end the pandemic. Preventing infection by developing T and B cell immunological memory is the goal of vaccination, while the development of immunological memory will protect against future diseases [4]. The research resulted in new vaccine technologies such as mRNA and DNA vaccines, some of which have never been tested in humans. Historically, most vaccines worked by inoculating non-harmful attenuated viral vectors, triggering an immune response. The antigen was either an inactivated infectious agent or a purified infectious agent protein. Because only the recognition of the SARS-CoV-2 genetic sequence is required, which was published by Chinese scientists on 11 January 2020, large viral DNA or RNA can be produced quickly [4,5]. Unlike classical vaccines, Pfizer BioNTech and Moderna’s modern vaccines contain the genetic information required to synthesize the SARS-CoV-2 spike protein, usually found on the viral surface. After receiving the vaccine, the spike protein is recognized by the immune system. Thus, mRNA is degraded in the cytoplasm and does not enter the nucleus, so it does not become part of the cell’s DNA [6,7]. They use a modified viral vector to deliver the SARS-CoV-2 spike protein into the cell, triggering an immune response.

According to the Romanian National Committee of Coordination of the Activities regarding Vaccination against COVID-19 (CNCAV), by 13 March 2022, more than 16.7 million doses of vaccine were administered, with above 8 million people fully vaccinated (2 doses) and only 2.5 million up to date (including the booster dose), which was initiated in September 2021.

Initially, pregnant women were excluded from the clinical trials of mRNA vaccines, although these have been demonstrated to be highly effective in the general population. Currently, the American College of Obstetricians and Gynecologists (ACOG), the Centre for Disease Control and Prevention (CDC), and the World Health Organization (WHO) recommend the vaccination of all eligible people, including pregnant women or those during the postpartum period [8,9].

This study confirms the importance and benefits of vaccinating against SARS-CoV-2 during pregnancy, with the development and transmission of spike protein antibodies both by placental passage to the fetus and via breastmilk to the newborn. It is safe to say that double protection is realized for both the mother and fetus dyad during pregnancy and for the mother and newborn dyad during the postpartum period. Blood collection was performed in the delivery room by venipuncture from the mother by umbilical cord blood collection from the newborn. Antibody assessment was realized by quantifying the level of maternal and neonatal SARS-CoV-2 receptor-binding domain (RBD) spike protein antibodies in the mother and her newborn, along with the predictors of spike protein antibody titers. It is not known precisely for how long the spike protein antibodies can persist in neonatal or infant blood, respectively.

## 2. Materials and Methods

### 2.1. Study Design and Settings

A single-center cohort study was conducted at the Première Hospital (Regina Maria Healthcare Network), Timisoara, Romania, over a period of 10 months (May 2021–February 2022) to quantify the serum and breastmilk values of SARS-CoV-2 spike protein antibodies in both the mother and her newborn, after complete vaccination during pregnancy. This study was approved by the Première Hospital’s Ethics Commission Board (No. 330/18.11.2021), as well as by the Ethics Committee of Scientific Research of “Victor Babeș” University of Medicine and Pharmacy Timișoara (No. 76/2020).

### 2.2. Participants

Mothers who were fully vaccinated with the mRNA vaccine (BNT162b2 Pfizer/BioNTech) against COVID-19 during pregnancy were included in the analysis. Pregnant women vaccinated with only 1 dose were excluded from the study. The “booster” dose (3rd dose) was not administered to either of the subjects because vaccination initiation for pregnant women began later on. Some of the vaccinated participants presented SARS-CoV-2 infection during pregnancy as well, but before vaccine administration. The percentage of vaccinated mothers during pregnancy was low in our clinic, representing only 7.9% of all births during the aforementioned period, this being possible because of late vaccination initiation among pregnant women (April–May 2021) and, on the other hand, because of a lack of strong evidence to suggest the vaccine’s impact onto the product of conception. Furthermore, large-scale vaccination studies regarding the safety and effectiveness of vaccines against SARS-CoV-2 during pregnancy would have been necessary.

### 2.3. Variables and Data Sources

Antibodies against the receptor-binding domain of the SARS-CoV-2 spike protein were quantified both in the maternal and neonatal serum after birth. Subsequently, spike protein antibodies were evaluated from breastmilk as well.

From the patient’s medical history, along with their written consent, data regarding gestational age, gravida/para, pregnancy-associated pathology, delivery type, APGAR score, anthropometric measurements at birth, SARS-CoV-2 infection during pregnancy, treatment in case of infection, moment of vaccine administration—first and second dose— (related to gestational age and administration protocol), symptoms after vaccination, spike protein antibody quantification in serum and breastmilk and neonatal evolution were collected.

### 2.4. Laboratory Analysis

In vitro quantitative determination of IgG antibodies against the RBD spike protein of SARS-CoV-2 in both serum and breast milk was performed by the electrochemiluminescence method using Cobas Elecsys e411 equipment (F. Hoffmann-La Roche AG, Basel, Switzerland).

The ECLIA immunohistochemistry method (electrochemiluminescence) uses a recombinant protein representing the RBD of the spike (S) antigen in a double-antigen sandwich type format, which favors the quantitative detection of high-affinity antibodies against SARS-CoV-2, with a total test duration of 18 min. The reactants used included streptavidin-coated microparticles, a biotinylated SARS-CoV-2 RBD domain as a recombinant antigen, and a ruthenium-marked SARS-CoV-2 RBD domain as a recombinant antigen.

The test is suggested as an adjuvant for evaluating the humoral immune reaction to SARS-CoV-2 spike protein. All instructions provided by the manufacturer were followed thoroughly, and the serum was collected in standard collection tubes with gel and no clot activator. The minimum detection limit, under which the result is negative for the presence of antibodies against the spike protein, represents 0.80 U/mL; above this value, the result is positive. No more than 2 mL of blood were drawn from the umbilical cord to determine the titer of RBD spike protein antibodies passed from the vaccinated mother to her newborn.

Neonatal values were compared to maternal serum values collected from venous blood. Blood immunological samples were drawn at almost the same moment for both the mother and newborn and were analyzed on the same day.

On the 3rd day of admission, samples of breastmilk were collected. Sampling was performed using a sterile technique by expressing 2 mL colostrum before the newborn’s first feed of the day. We decided not to deprive the newborn of breastmilk during the first 2 days of life when the quantity of milk is reduced, so the sampling was performed during the 3rd day of life.

### 2.5. Statistical Analysis

IBM SPSS v.22 (IBM Corp., Armonk, NY, USA: IBM Corp., Armonk, NY, USA) was used to conduct all analyses. There were no missing values, except for one value of breastmilk antibodies (*Ab milk*), due to lactation suppression. Because we used *t*-tests and regression in our analyses, we first checked the assumptions of these tests. There were no serious deviations from the assumption of normality for any of the continuous variables investigated, except for *Ab milk* and gestational age (in the subsample of mothers who did not have COVID-19), which had kurtosis values of 5.85 and 5.06, respectively. However, these values are within a more relaxed criterion of normality proposed by Hair et al. [10], who consider data consistent with the assumption of normality if skewness values are between −3 and 3 and kurtosis values are between −7 and 7; therefore, the following analyses were retained. Homoscedasticity was investigated by inspecting the normality of residuals for all models used. No violations were observed for either skewness or kurtosis. Therefore, we retained all variables in our analyses.

To investigate whether antibodies from the mother are transmitted to the child before and after birth, it was first assessed whether detected antibodies from the newborn’s blood and mother’s milk were significantly higher than 0.

## 3. Results

A total of 91 mother/newborn dyads were included in the study. All participants in the study had detectable SARS-CoV-2 RBD spike protein antibodies both in serum and in breastmilk. Maternal and neonatal serum values were above the detection limit (>0.80 U/mL), with the highest maternal values being 19,523 U/mL (5077 ± 5733 SD), and neonatal ones 18,765 U/mL (5288 ± 5661 SD). The average duration between vaccination and delivery was 15.2 weeks, since 44 (48.4%) women were vaccinated in the third trimester, 27 (29.7%) in the second trimester, and the other 20 (22.0%) pregnant women received the COVID-19 vaccine during the first trimester.

**Figure 1:**
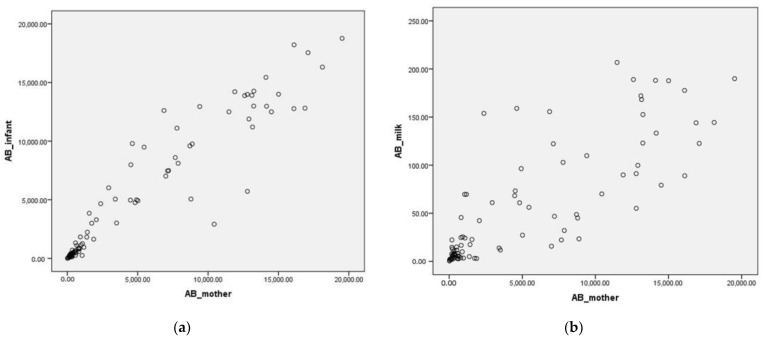
Correlation graph between: (**a**) neutralizing anti-SARS-CoV-2 spike (S) protein antibodies in maternal serum and neonatal serum; The *x*-axis shows the maternal antibody titer and the *y*-axis the neonatal antibody titer. (**b**) neutralizing anti-SARS-CoV-2 spike (S) protein antibodies in maternal serum and breastmilk; The *x*-axis shows the titer of maternal antibodies and the *y*-axis the titer of breast milk antibodies.

A Pearson correlation test revealed very high and significant correlations between maternal and neonatal antibodies, *r* = 0.95, *p* < 0.001, and between maternal serum and breastmilk antibodies, *r* = 0.82, *p* < 0.001, reinforcing the idea that antibodies are transferred from the mother to her child and indicating that mothers who produce a stronger immunological response also provide better protection for their offspring (Figure 1).

From the studied group of vaccinated women during pregnancy, 26 patients associated with COVID-19 during the first or second trimester of pregnancy but before vaccination. In this situation, the decision to vaccinate was taken during the third trimester of pregnancy, according to the recommendations of their obstetrician. In these cases, the patients presented a variety of symptoms, from fever, shivers, anosmia, and ageusia, up to fatigability, with no further associated complications. Treatment consisted of antipyretics, anticoagulants, and vitamins, with strict monitoring performed by their obstetrician and family doctor at home, with favorable evolution in all cases.

Post-vaccination symptoms were limited to pain at the site of injection. Only one case had generalized muscle pain 24 h after vaccination with the second dose, with favorable evolution.

Therefore, in our sample there were both vaccinated mothers who did not have COVID-19 (*n* = 65) and vaccinated mothers who had the disease (*n* = 26). The immunological response was significantly higher for mothers who also had the disease, in the matter of serum antibodies (mean difference = 9146.52; *t*(89) = 9.92, *p* < 0.001), which was also reflected in the number of antibodies detected in neonatal serum(mean difference = 8577.14, *t*(89) = 8.95, *p* < 0.001) and the number of antibodies detected in mother’s milk (mean difference = 83.91; *t*(88) = 7.56, *p* < 0.001) (Table 1).

As a next step, in a regression analysis, we investigated what exactly predicts the number of antibodies in maternal serum (i.e., *Ab. mother*), in neonatal serum, and in the mother’s own milk. For mothers who did not have COVID-19, *Ab. mother* was significantly predicted by the vaccination trimester (*b* = 971.91, *β* = 0.24, *t*(60) = 1.95, *p* = 0.028) but not by the mother’s age (*b* = −160.25, *β* = −0.21, *t*(60) = −1.62, *p* = 0.111), para (*b* = −50.42, *β* = −0.01, *t*(60) = −0.07, *p* = 0.945) or autoimmune thyroiditis (*b* = −14.91, *β* = −0.00, *t*(60) = −0.01, *p* = 0.989), indicating that the amount of antibodies in maternal serum increases with 971.91 for each increasing trimester of vaccination, while mother’s age, para or mother’s autoimmune thyroiditis had no significant influence on the number of antibodies. These predictors explained about 10.4% of the variability in *Ab. mother* (R^2^ = 0.104). For mothers who had COVID-19, AC mother was not significantly predicted by any of these variables (Table 2).

**Figure 2:**
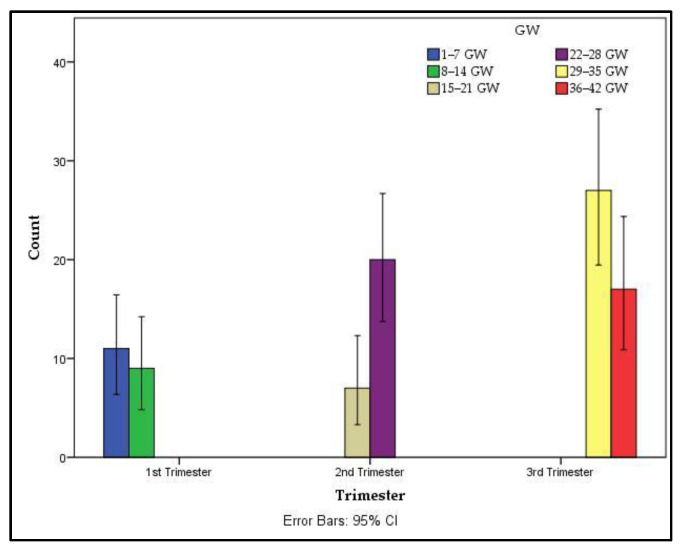
Distribution of vaccination with Pfizer/BioNTech based on the trimester of pregnancy. The *x*-axis represents the trimester of pregnancy/gestational weeks and the *y*-axis represents the count of vaccinated pregnant women; GW—Gestation Weeks.

Figure 2 describes the distribution of BNT162b2 Pfizer/BioNTech vaccine administration related to gestational age. Every pregnant woman included in the study benefited from a complete immunization scheme (2 doses). Vaccination occurred during pregnancy, with strict protocol following regarding the second dose administration, which was 3 weeks after the first dose. Maternal COVID-19 infection was not present in the interval between vaccination and birth. None of them managed to obtain the booster shot due to the late immunization initiation of pregnant women and the short time interval of the study (May 2021–February 2022).

We could observe that pregnant women who associated COVID-19 during pregnancy, along with a complete immunization schedule against SARS-CoV-2 during the gestational period, transmitted higher antibody titers to their newborn compared to pregnant women who did not have the disease. The mean neonatal antibody value for those born to mothers who had COVID-19 can be observed in Table 1 and was 11,414.90 U/mL, while the ones born to mothers who were only vaccinated were 2837.76 U/mL.

In 87 cases, the birth took place at term, while four were born preterm, but with gestational ages above 34 weeks. APGAR score ranged between 8 and 10, all having an appropriate neonatal adaptation. A single case presented with severe obstetrical asphyxiation, having an APGAR score of one at 1 min of life, five at 5 min, and seven at 10 min, with favorable outcomes after neonatal resuscitation. Anthropometric measurements have shown a mean birthweight of 3331.6 g (SD = 417.7), a mean height of 50.7 cm (SD = 1.5), and a mean head circumference of 34.54 cm (SD = 1.3).

For mothers who did not have the disease (COVID-19), the antibodies in infants serum (*Ab infant*) were predicted by vaccination trimester (*b* = 1032.60, *β* = 0.23, *t*(59) = 1.80, *p* = 0.039) and maternal age (*b* = −252.71, *β* = −0.29, *t*(59) = −2.17, *p* = 0.034) but not by gestational age (*b* = −339.12, *β* = −0.10, *t*(59) = −0.80, *p* = 0.427), type of birth (*b* = 343.53, *β* = 0.04, *t*(59) = 0.32, *p* = 0.750) or para (*b* = −41.30, *β* = −0.01, *t*(59) = −0.05, *p* = 0.961), indicating that the titer of antibodies in infant’s serum increases with 1032.60 for every increasing trimester of vaccination and decreases with 252.71 for every increasing year in mother’s age, while gestational age, type of birth and para seem to have no significant influence. These predictors explained about 12.7% of the variability in *Ab infant* (R^2^ = 0.127). For those mothers who had COVID-19, *Ab infant* was not significantly predicted by any of the studied predictors (i.e., all *p* > 0.05).

In our study, 32 mothers presented associated pathologies, 11 of them suffering from Hashimoto’s autoimmune thyroiditis (Table 3). We observed that the associated pathology did not influence immunological response after vaccination for these patients.

During the third day of hospitalization, we collected breastmilk samples that were tested using the same ECLIA method for the quantification of SARS-CoV-2 RBD spike protein antibodies. All breastmilk samples were positive for antibodies, which leads to double neonatal protection against SARS-CoV-2, as long as breastfeeding is encouraged and the mother is provided with all needed information. The highest detected value was 206.7 U/mL.

Regarding the variability of antibodies in mother’s own milk, for those who did not have COVID-19, *Ab milk* was predicted by *vaccination trimester* (*b* = 13.60, *β* = 0.24, *t*(58) = 1.95, *p* = 0.028) but not by *mother’s age* (*b* = −1.81, *β* = −0.17, *t*(58) = −1.25, *p* = 0.216), *gestational age* (*b* = −2.13, *β* = −0.05, *t*(58) = −0.41, *p* = 0.681), *type of birth* (*b* = 22.94, *β* = 0.23, *t*(58) = 1.76, *p* = 0.084) or *para* (*b* = −3.40, *β* = −0.04, *t*(58) = −0.33, *p* = 0.743), indicating that the number of antibodies in maternal breastmilk increases with 13.60 for each increasing trimester of vaccination, while mother’s age, gestational age, type of birth or para have no influence on the amount of antibodies in mother’s milk. These predictors explained about 14.3% of the variability in *Ab milk* (*R*^2^ = 0.143). Again, for those mothers who had the disease, *Ab milk* was not significantly predicted by any of the studied predictors.

## 4. Discussion

This study aimed to demonstrate if the vaccination against SARS-CoV-2 during pregnancy offers important neonatal protection by transplacental and breastmilk antibody passage, leading to a significant risk reduction for this category, correlated with strict adherence to the current epidemiological measures. As previously determined in other studies, vaccination efficacy using the Pfizer/BioNTech vaccine rises to 81% regarding the protection against symptomatic COVID-19 among the general population [11], and the extent this vaccine can be used was determined as safe during pregnancy in a large cohort of more than 35 thousand pregnant women [12]. Pregnant women are at high risk of progressing to moderate and severe COVID-19, with developing pregnancy-induced hypertension, associated bacterial infections that require antibiotherapy, and a higher admission rate in intensive care units [13]. For these reasons, ACOG and CDC recommend vaccination against SARS-CoV-2 during pregnancy.

The women included in the study decided to vaccinate together with their obstetrician, who thoroughly explained the benefits of vaccination along with fetal immunity induction once immunization is accomplished. The patients had no associated risk factors that could advise against vaccination. In a study conducted by Skjefte M et al., the level of vaccine acceptability amongst pregnant women was around 52% [14]. In a systematic review led by Januszek S et al., over 33 studies that appealed the acceptability of SARS-CoV-2 vaccination for pregnant or lactating individuals was between 29.7 and 77.4% [15]. In our country, a study conducted by Citu IM et al. concerning vaccine acceptability for pregnant women observed a hesitancy of 52.5% vs. 40.3% for non-pregnant individuals [16].

In our study, 100% of participants presented SARS-CoV-2 RBD spike protein antibodies in both maternal and neonatal serum, as well as in breastmilk. These results suggest that antibodies from the mother are transmitted to the child using two different pathways: through the umbilical during intrauterine life and human milk after birth. Prabhu et al. showed that the percentage of antibodies found in the neonatal umbilical cord serum born to vaccinated mothers was 99% [17]. Omer et al. describe higher antibody titers in both serum and breastmilk compared to COVID-19 infected and recovered mothers [18]. Furthermore, another study conducted in Romania showed that although peak antibody titers decrease steadily and halve after only 4 months, and IgG response is significantly higher in seropositive individuals, there is unquestionable positive immunogenicity of the BNT162b2 vaccine against the most commonly circulating SARS-CoV-2 viral strains in the pregnant population [19].

The SARS-CoV-2 RBD spike protein antibody titers described in our study for both mothers and newborns are similar to the ones described by Kugelman N et al., which suggested all maternal and newborn SARS-CoV-2 IgG tests had positive results. The median level of IgG antibodies at birth was 1185.2 AU/mL (range, 146.6–32,415.1 AU/mL) for parturient women and 3315.7 AU/mL (range, 350.1–17,643.5 AU/mL) for neonates, with neonatal titers measuring approximately 2.6 times higher than maternal titers [20]. We observed that in most situations, neonatal serum values were higher than the serum values of their mothers, mostly because of the active and passive antibody transfer from the mother to her child, but also possibly due to the prolonged antibody persistence in the neonatal blood, with slow, secondary degradation.

Before 16 weeks gestational age, the antibody transfer is minimal, with an increase during the second trimester of pregnancy, reaching a peak during the third trimester, especially during the last 4 weeks of pregnancy. This can explain higher antibody values in newborns versus mothers [21,22,23]. Nevertheless, it was previously determined that newborns do not seem to be at higher risks of COVID-19 if born from mothers positive for SARS-CoV-2; therefore, breastfeeding, close contact, or in utero infection do not increase the risk of infection for infants [24]. In our study, many situations show higher neonatal values compared to their mothers, on the one hand, due to the active and passive antibody transfer taking place from the mother to her fetus and newborn and, on the other hand, due to a possible longer persistence of antibodies in neonatal circulation, with gradually slower secondary degradation [20,23,25]. Charepe N et al. described higher IgG antibodies in patients who breastfed for a longer period, leading to reassurance in breastfeeding continuation beyond 6 months of age [25].

The quantity of spike protein antibodies detected in maternal serum, neonatal serum, and breastmilk, respectively, was highly influenced by the time between vaccination and birth. The mothers vaccinated during the first trimester of pregnancy, and their resulting newborns had the lowest antibody values in both serum and breastmilk, indicating that newborns might better benefit from maternal antibodies if vaccination occurs during the third trimester of pregnancy. Even though all newborns had detectable serum antibodies, the protective titer is not known for this category. Ulterior studies are required to establish these facts, relating to the fact that neonatal antibody elimination is different from adults. The protection realized by the mother for her newborn usually lasts for 6 to 12 months [26].

Shook L et al. discovered the percentage of newborns from vaccinated mothers who had detectable serum SARS-CoV-2 IgG spike protein antibodies was around 94% by 2 months of age and 60% by 6 months of age. Surprisingly, only 8% of those born to mothers infected with SARS-CoV-2 but without vaccine during pregnancy had detectable antibodies, a fact that suggests strong vaccine-induced protection [27].

A study conducted for 8 months by Lipkind HS et al. addressing the incidence of prematurity and early gestational age amongst newborns from mothers vaccinated with mRNA vaccine during pregnancy compared to non-vaccinated women demonstrated no significant correlation between the two (*p* = 0.06), so vaccination against SARS-CoV-2 must be encouraged [28].

Associated pathologies had no influence over the immunological response of pregnant women to SARS-CoV-2 vaccination during pregnancy, Hashimoto’s autoimmune thyroiditis being present in 11 cases. Paschou SA et al. provided evidence that patients with autoimmune thyroiditis present a similar immunological response to the COVID-19 BNT162b2 mRNA vaccine (Comirnaty, Pfizer/BioNTech) with healthy subjects [29].

The above-mentioned cases studied in the current paper show that SARS-CoV-2 RBD spike protein antibodies acquired by vaccination are transferred transplacentally from the mother to her fetus, with continuation after birth by trans-mammary passage via breastmilk.

Our study could not follow up the SARS-CoV-2 RBD spike protein antibody assessment after patient discharge due to a lack of maternal compliance who did not return to the medical office for the neonatal check-up by 1 month of life, according to the Hospital’s appointment or refused blood or breastmilk sampling. However, out of 91 mother/newborn dyads, 12 accepted blood and breastmilk collection, and the results showed persistence of antibodies, a fact that will be addressed later on within a significant study group. Furthermore, it is important to mention that we encourage pregnant women to receive a booster dose as well if more than six months have passed since the last vaccine, as antibody titers tend to decrease over time. In the current study, almost half of the patients were vaccinated during the third trimester of pregnancy. Therefore, the antibody titer measured at delivery might be higher than expected.

## 5. Conclusions

During COVID-19 pandemics, pregnant women with or without preexistent pathology or pregnancy-induced pathology represent a risk for the mother/fetus dyad. Vaccinating pregnant women seemed an optimal solution to disease prophylactics and protection of both the fetus and the newborn.

Our study, conducted on 91 mother/newborn dyads, demonstrated, beyond question, that the vaccine administration in different periods of pregnancy induces antibody production required for the possible protection of both the mother and her child, which can be further assessed. The period between vaccination and birth influenced the titer of serum and breastmilk antibodies.

Consequently, this study shows the importance of maternal vaccination against SARS-CoV-2 in terms of neonatal benefits from the antibody passage via the placenta during intrauterine life and breastmilk after birth. This double protection can be achieved by encouraging breastfeeding, bonding, and providing maternal empowerment to participate in her infant’s care.

Further studies are required to determine the protective neonatal/infant antibody values and the duration of this protection, along with active vaccination campaigns for pregnant women, emphasizing the benefits of vaccination during pregnancy.

## Figures and Tables

**Table 1:** Descriptive statistics of the cohort of patients.

	No COVID-19 (*n* = 65)	COVID-19 (*n* = 26)	*t* (*p* Value)	Overall (*n* = 91)
	Mean ± SD (95% CI)	Mean ± SD (95% CI)		Mean ± SD (95% CI)
*Ab. mother* (U/mL)	2463.92 ± 3267.3 (1654.32–3273.52)	11,610.44 ± 5373.40 (9440.08; 13,780.80)	9.92 (*p* < 0.001)	5077.21 ± 5733.64 (3883.12–6271.30)
*Ab. infant* (U/mL)	2837.76 ± 3711.8 (1918.01–3757.52)	11,414.90 ± 5046.56 (9376.55; 13,453.25)	8.95 (*p* < 0.001)	5288.37 ± 5661.50 (4109.31–6467.44)
*Ab. milk* (U/mL)	30.98 ± 45.38 (19.64–42.31)	114.89 ± 53.27 (93.37; 136.40)	7.56 (*p* < 0.001)	55.22 ± 60.97 (42.45–67.99)

Notes: *Ab. mother* = antibodies present in maternal serum, *Ab. infant* = antibodies present in neonatal serum, *Ab. milk* = antibodies present in breastmilk.

**Table 2:** Regression analysis.

	No COVID-19 (*n* = 65)			COVID-19 (*n* = 26)		
DV/Predictors	*b*	*β*	*p*-Value	*b*	*β*	*p*-Value
*Ab. mother*						
Vaccination trimester	971.91	0.24	0.028 *	−989.78	−0.15	0.751
Mother’s age	−160.25	−0.21	0.111	188.51	0.15	0.476
Para	−50.42	−0.01	0.945	−2663.58	−0.29	0.219
Autoimmune thyroiditis	−14.91	−0.00	0.989	−1996.32	−0.12	0.574
*Ab. infant*						
Vaccination trimester	1032.60	0.23	0.039 *	−860.04	−0.14	0.719
Mother’s age	−252.71	−0.29	0.034 *	116.69	0.10	0.642
Gestational age	−339.12	−0.10	0.427	426.04	0.10	0.652
Type of birth	343.53	0.04	0.750	−3063.59	−0.30	0.254
Para	−41.30	−0.01	0.961	−3919.06	−0.45	0.092
*Ab. milk*						
Vaccination trimester	13.60	0.24	0.028 *	−16.97	−0.27	0.859
Mother’s age	−1.81	−0.17	0.216	2.32	0.18	0.384
Gestational age	−2.13	−0.05	0.681	−3.15	−0.07	0.752
Type of birth	22.94	0.23	0.084	−6.80	−0.06	0.807
Para	−3.40	−0.04	0.743	−25.54	−0.28	0.287

Notes: *Ab. mother* = antibodies present in maternal serum, *Ab. infant* = antibodies present in neonatal serum, *Ab. milk* = antibodies present in breastmilk; *b* = unstandardized coefficients, *β* (beta) = standardized coefficients, * = statistically significant.

**Table 3:** Associated pathologies during pregnancy.

Pregnancy Associated Pathologies and Complications	Number of Cases
Pregnancy-induced hypertension (PIH)	2
Gestational diabetes	2
Hashimoto’s autoimmune thyroiditis	11
Hypothyroidism	4
Pituitary microadenoma	1
AgHBs carrier	3
Thrombophilia	5
Preterm birth	4

## Data Availability

Not applicable.
